# Inexpensive, serotype-independent protocol for native and bioengineered recombinant adeno-associated virus purification

**DOI:** 10.14440/jbm.2016.102

**Published:** 2016-05-03

**Authors:** Erik Arden, Joseph M. Metzger

**Affiliations:** Department of Integrative Biology & Physiology, University of Minnesota Medical School, Minneapolis, MN 55455, USA

**Keywords:** adeno-associated virus, purification, PEG8000, centrifugation, inexpensive

## Abstract

Recombinant adeno-associated virus (AAV) is a valuable and often used gene therapy vector. With increased demand for highly purified virus comes the need for a standardized purification procedure that is applicable across many serotypes and includes bioengineered viruses. Currently cesium chloride banding or affinity chromatography are the predominate forms of purification. These approaches expose the final purified virus to toxic contaminants or are highly capsid dependent and may require significant optimization to isolate purified AAV. These methods may also limit crude viral lysate processing volume resulting in a significant loss of viral titer. To circumvent these issues, we have developed an AAV purification protocol independent of toxic compounds, supernatant volume and capsid moiety. This purification method standardizes virus purification across native serotype and bioengineered mosaic capsids.

## BACKGROUND

Recombinant and pseudotyped adeno-associated virus (AAV) is the vector of choice for many laboratories because of its ability to maintain *in vivo* gene expression throughout an animal’s life span. Naturally isolated serotypes 1-9 have been the most intensely studied and used in the laboratory setting. Each of these capsids displays a significantly different tissue tropism panel, although none appear to be target specific [1-4]. These off-target effects can dilute transduction and manifest confounding results. These observations have led investigators to successfully engineer mosaic capsids that refine the vectors targeting ability [5].

One significant hurdle for incorporating AAV into laboratory experiments remains rapid and facile vector purification across many serotypes. Current purification methods generally involve gradient centrifugation or affinity column purification. Cesium chloride and iodixanol gradients are used in many laboratories as they are independent of capsid binding moieties. While gradient purification is useful, it has been shown to significantly reduce infectivity. In addition, the associated health risks and toxicity may introduce confounding experimental results if not completely removed. Affinity column chromatography is also a favored method of vector isolation. This method relies on capsid binding motifs and often requires significant resources to optimize. However, considering the massive potential for capsid design and manipulation, purification techniques requiring the presence of a specific moiety are simply not practical.

Here, we detail an inexpensive purification method independent of gradient centrifugation, capsid moiety and has been used by us with success for high titer (>2 × 10^13^ vector genomes [vg]/ml) viral-mediated *in vivo* heart transduction studies [6]. This protocol incorporates polyethylene glycol 8000 precipitation of empty and full AAV capsids and two spin purification steps based on the sedimentation coefficient for AAV2 capsids. The sedimentation coefficients of empty and full capsids are ~72 s and ~138 s respectively. As proof-of-concept, we used our protocol to purify pseudotyped AAV2/6 and AAV2/41. AAV2/41 is a mosaic capsid created through DNA shuffling experiments and contains elements from capsids AAV 1, 6, 7, and 8 [11].

While several previously published protocols for AAV purification incorporate PEG to precipitate virus [13], all focus on cell pellet processing and discard culture media at harvest. In our experience, this translates into a significant loss of titer (upwards of 80%) due to HEK cells detaching during viral production. For this reason, we developed our protocol to capture AAV virions in both cell pellet and cell media concomitantly. Transfecting adherent HEK293 cells is still the most popular method of AAV production and we have developed our protocol to reflect primary research (non-clinical) needs.

All viruses contain a constitutively expressed luciferase cassette. The protocol is focused on a rapid and inexpensive rAAV production/purification technique that offers several benefits over existing methods. These include ease of use by incorporating Cell Factory transfection, whole preparation processing reducing loss of titer, high titers, no gradient or chloroform extraction required over existing protocols [7-10]. This protocol is written here to be highly accessible to the non-specialist.

## MATERIALS

### Reagents

• HEK293 Cells (ATCC CLR-1573)
• Dual or Tri-Plasmid AAV System (Chamberlain [1])
• Dulbecco’s Modified Essential Media (Life Technologies 11995-073)
• Fetal Bovine Serum (HyClone SH30071.03)
• L-Glutamine (100 ×) (Life Technologies 25030-081)
• Antibiotic-Antimycotic (100 ×) (Life Technologies 15240112)
• MEM Non-Essential Amino Acids (100 ×) (Life Technologies 11140-050)
• 200 mM Magnesium Chloride (Sigma M8266)
• Giga-Scale Plasmid Purification System (Qiagen 12991)
• PBS (1 ×) (HyClone SH30256)
• Benzonase Endonuclease (Sigma E1014-25KU)
• Protease Inhibitor (100 ×) (Sigma P2714)
• RNAse A (Sigma R4875)
• Tris Acetate-EDTA (10 ×) (Sigma T8280)
• Calcium Chloride Hexahydrate (Sigma 442909)
• HyClone Water, Molecular Biology Grade (Thermo Scientific SH30538)
• N,N-Bis(2-hydroxyethyl)-2-aminoethanesulfonic Acid (BES) (Sigma B4554)
• Sodium Phosphate Dibasic (Sigma S3264)
• Sodium Chloride (Sigma S5886)
• Polyethylene Glycol 8000 (PEG8000) (Fisher Scientific BP233-1)

### Recipes

All cell culture, transfections, related media handling and the final viral suspension should be performed in a biosafety cabinet using sterile technique. Plasmid preparations, lysate filtrations and centrifuge tube transfers may be performed at the bench.

#### Cell culture media

For For cell seeding media, add 100 ml FBS, 10 ml L-Glutamine, 10 ml Antibiotic-Antimycotic and 10 ml 200 mM MgCl_2_ to 870 ml DMEM. The serum exchange media is DMEM supplemented with L-Glutamine, Antibiotic/Antimycotic, 1x MEM Non-Essential Amino Acids only.

#### Plasmid DNA

Plasmid DNA should be prepared previous to transfection and stored at -20^°^C for up to one year. The most efficient method of preparing large amounts (5–10 mg) is by using giga-scale purification kits. Qiagen provides an extremely fast, pure and high concentration DNA stock in their Plasmid Plus Giga Kit. Plasmid stocks should be suspended to 1.0 mg/ml.

1 **Tip**: Plasmid DNA must have a 260 nm/280 nm absorbance ratio between 1.80 and 1.85. Plasmid stocks that fall out of this range cause extremely large aggregates during transfection that are not readily taken up by cells. In addition, all plasmids should be maintained in *E. coli* strains engineered for low recombination events, as the AAV2 ITR’s display significant recombination with the bacterial genome. To ensure minimal recombination, ITR’s should be digested as described previously [12,13].

#### Calcium phosphate precipitation reagents

*Transfection Reagent A.* Transfection reagent A is made up fresh at the time of transfection. In a 250 ml conical tube combine 50.4 ml ddH_2_O, 6.0 ml 2.5 M CaCl_2_ and AAV plasmids. Our dual plasmid system uses 1.2 mg shuttle vector to 2.4 mg pDGM6.

*Transfection Reagent B.* Transfection reagent B can be made up previously and stored at room temp for up to six months. Combine 50 mM BES, 1.5 mM Na_2_HPO_4_ and 280 mM NaCl_2_.

**Tip**: pH must be between 7.00 and 7.05.****

#### 10 × citric saline cellular detachment solution

• 1.35 M KCl
• 0.15 M sodium citrate

For a 1 × working stock, dilute 20 ml 10 × solution to 200 ml in Hyclone molecular biology grade water.

#### Precipitation reagent stock solution (PEG/NaCl) (5 ×)

This precipitation solution should be made in advance and can be stored at room temp for up to three months. Dissolve 400 g PEG8000 and 146.1 g NaCl_2_ in 1.0 L ddH_2_O.

#### Pellet suspension buffer

Dissolve 14.61 g NaCl in 1.0 L Hyclone Water for a final concentration of 250 mM.

### Animals

C57Bl6 mice were used in this study to determine in vivo functionality of purified vector. All experiments were in complete compliance with Institutional animal guidelines.

### Equipment

• Forma Steri-Cult CO_2_ Incubators (Thermo Scientific 3311) (Humidified, 5.0% CO_2_ regulated)
• Microfluidizer (Microfluidics M-110L Pneumatic)(Chilled on wet ice, 200micron Lysis Chamber, 9000psi Chamber Pressure)
• Li-Cor Odyssey Imager (Li-Cor)
• High Performance Centrifuge (Beckman Coulter J Series)
• JA-14 Rotor (Beckman #339247)
• Ultra-Centrifuge (Beckman Coulter Optima Series)
• SW-32 Rotor (Beckman Coulter)
• Ultra-Clear Thin-Walled Centrifuge Tube (Beckman Coulter 344058)
• Class II Bio-Safety Cabinet (Thermo Scientific EN12469)
• Temperature Controlled Water Bath (Fisher Isotemp 15-462-10Q)
• Tissue Culture Table Top Centrifuge (Sorval Legend RT 75004377)
• Swinging Bucket Rotor (Sorval 75006445)
• 500 ml Bucket Adaptor (Heraeus 75006441L)
• Bottle Rotor Cushions/Adaptors (Corning 431124)
• 10 Tray Cell Factory (Nunc EasyFill 140400)
• 1 Tray Cell Factory (Nunc EasyFill 140000)
• 150 ml 0.22 µm Express Plus Steritop Filter Unit (Millipore SCGPT01RE)
• 1000 ml Stericup Receiver Flask (Millipore SC00B10RE)
• 50 ml Conical Tube (Corning 430291)
• 250 ml Conical Tube (Corning 430776)
• 500 ml Conical Tube (Corning 431123)

## PROCEDURE

1. Cell Factory seeding and calcium phosphate transfection
It is very important to assess cell confluency, precipitate size and transfection efficiency in the Cell Factory under magnification. However, because ten tray cell factories (abbreviated henceforth as 10TCF) are not easily visualized under magnification, we have incorporated a one tray Cell Factory (1TCF) as an observation control. It should be noted a highly efficient transfection will cause 60–80% cell detachment at time of harvest. For this reason, it is critical to process both culture media and cell pellet.
  1.1. Prepare 2.4 × 10^8^ HEK293 cells in 1.0 L 37^°^C Cell culture seeding media. Pipette 90 ml cell suspension into the 1TCF.
  1.2. Pour the remaining cell suspension into the 10TCF. Evenly distribute cell suspension across all layers.
  1.3. Incubate cell factories overnight at 37^°^C/5.0% CO_2_.
  1.4. Ensure Cell Factory monolayer is at optimal 70% confluence.
  1.5. Combine Transfection Reagent A solutions to a final volume of 60 ml.
  1.6. In a second tube, aliquot 60 ml of Transfection Reagent B.
  1.7. Add Transfection Mix A to Transfection B.
  1.8. Incubate CaPO_4_ mixture for five to thirty minutes at room temp. Precipitate formation should be monitored by visualizing 500 ml aliquots under 40 × magnification collected at 5–10 min intervals throughout the incubation. The mix should be applied to cells when it is visually very opaque and precipitate appears as fine granules under magnification.
  1.9. Pipette 10 ml transfectant into the 1TCF. Immediately pour the remaining mix into the 10TCF and distribute evenly across layers. Return to incubator and incubate between 16 h and 20 h.
  1.10. Gently remove cell factories from incubator and decant media/transfection mix. Ensure minimal cell loss occurs.
  1.11. Replace transfectant media with 800 ml 37^°^C Cell Culture Serum Exchange Media in the 10TCF and 80 ml in the 1TCF. Evenly distribute media across layers.
  1.12. Return cell factories to incubator for 48 h.
2. Cell and Media Harvest
  2.1. Remove cell factories from incubator and pool both into a 1.0 L collection flask.
  2.2. Detach remaining cells by applying 50 ml 2x citric saline detachment solution to 10TCF. Evenly distribute across trays and incubate with rocking at room temp for 5–10 min. Use 5.0 ml for the 1TCF.
  2.3. Visualize by eye cells have detached and pour into pooled supernatant receiver flask. Rinse Cell Factory three times with 50 ml unsupplemented DMEM.
  2.4. The viral supernatant may be processed immediately or stored at -80^°^C until purification.
3. Crude lysate processing
  3.1. Remove viral supernatant from freezer and thaw in 3.0 L beaker to catch any flask leakage. Viral lysate placed at ~25^°^C will thaw completely in 20 h.
  3.2. Chill a microfluidizer on wet ice previous to cell lysis. Run crude supernatant through microfluidizer according to manufactures instructions.
  3.3. Collect viral lysate in new 1.0 L Stericup Receiver Flask. Rinse fluidizer with 50 ml DMEM.
  3.4. Vacuum filter viral lysate through 150 ml 0.22 µm Express Plus Steritop Filter Unit into one 1000 ml Stericup Receiver Flask used for collection. The filter may need to be changed up to ten times.
**Tip**: The number of filter units used is proportional to the amount of cellular debris in the crude lysate. In our experience, highly efficient transfections contain less cellular debris and so require fewer filter units.
  3.5. Add 10 ml Protease Inhibitor Cocktail, 10 U/ml Benzonase and 10 mg/ml RNase A to the clarified viral lysate. Incubate for 3 h in 37^°^C water bath.
  3.6. Perform a second filter clarification identical to step 3.4. This clarification step should only require a few filter flasks.
4. Precipitation and purification
  4.1. Add 250 ml 1 × Precipitation Reagent Stock Solution to lysate containing flask and incubate on ice for 90 min.
  4.2. Transfer crude lysate/PEG mixture into two 500 ml conical tubes. The tubes can accommodate a maximum volume of ~650 ml (Crude Lysate in **Fig. 2**).
  4.3. Spin in bench top centrifuge at 3000 × g 4^°^C for 30 min.
  4.4. Remove and discard aqueous layer ensuring minimal pellet disruption (Aqueous Layer A in **Fig. 2**).
  4.5. Resuspend crude viral pellet in 40 ml Pellet Suspension Buffer and aliquot into 50 ml conical.
  4.6. Rinse tubes twice with Pellet Suspension Buffer and combine with pellet suspension (Crude Suspension in **Fig. 2**).
  4.7. Clarify pellet suspension by centrifugation at 10,000 × g and 4^°^C for 10 min.
  4.8. Transfer the viral containing aqueous layer to a Beckmann Ultra Clear SW32 tube and spin at 149,000 × g/4^°^C for 3 h. Discard aqueous layer ensuring minimal pellet disruption (Aqueous Layer B in **Fig. 2**).
  4.9. Resuspend pure viral pellet in 5.0–10.0 ml Pellet Suspension Buffer (Purified AAV in **Fig. 2**).

**Figure 1.**
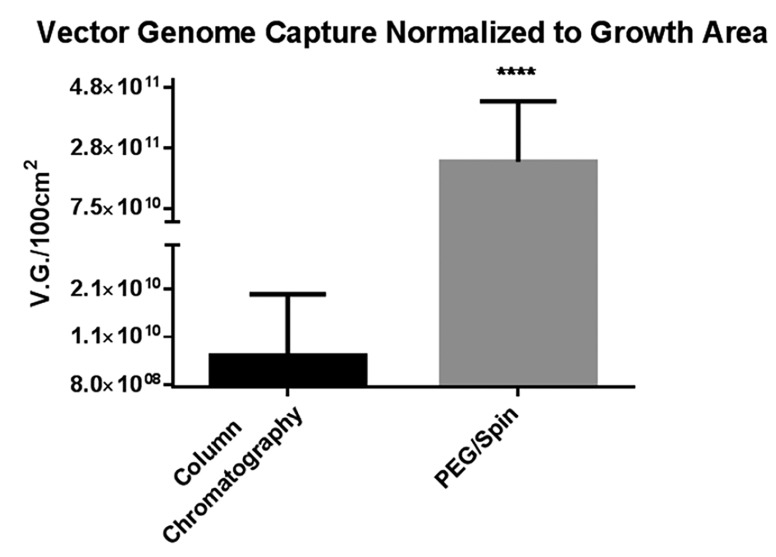
**Comparison of purified AAV2/6 vector genome captured using heparin column chromatography and our PEG/Spin protocol.** Using identical transfection and clarification procedures, the PEG/Spin captured on average 30 x more packaged genome as determined by qRT-PCR.

**Figure 2.**
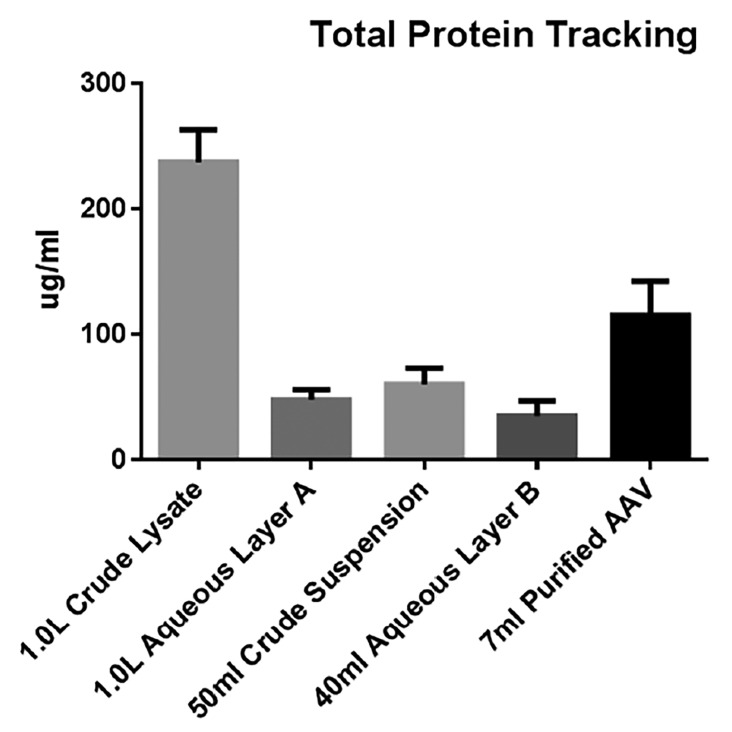
**Total protein tracking and capture through centrifugations and final pellet suspension.** Fractions collected as 1.0 ml aliquots. Aqueous layer A is PEG/NaCl treated supernatant immediately following 3000 × g centrifugation. Crude Suspension represents the PEG/NaCl pellet resuspended in 50 ml 250 mM NaCl prior to 10,000 × g centrifugation. Aqueous Layer B represents the suspension buffer immediately following the final 149,000 × g centrifugation. AAV pellet is suspended in 7.0 ml 250 mM NaCl.

**Figure 3.**
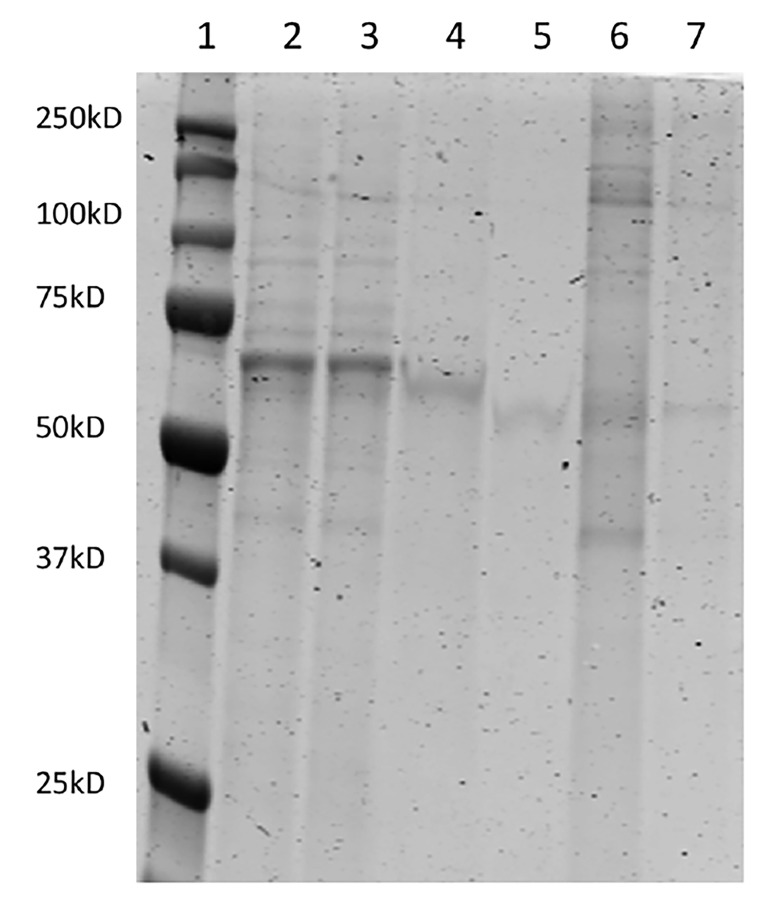
**SDS-PAGE electrophoresis on 20 microliter aliquots taken at different stages of purification.** Lane: (1) Marker (Bio-Rad Precision Plus), (2) Crude supernatant pre-enzymatic digest, (3) Crude supernatant post filtration, (4) PEG/NaCl treated supernatant pre-3,000 × g centrifugation, (5) Aqueous layer post 3,000 × g centrifugation, (6) Crude PEG/NaCl pellet suspended in 40 ml Suspension Buffer pre-10,000 × g spin, (7) Virus containing aqueous layer post 10,000 × g spin.

## ANTICIPATED RESULTS

We developed a new AAV purification protocol and validated it using pseudotyped AAV2/6 and AAV2/41. AAV2/41 displayed similar results to AAV2/6 (data not shown). Figure one compares AAV2/6 heparin sulfate column and our PEG/Spin method based on vector genome recovery normalized to cell growth area.

A 1.0-L AAV Cell Factory transfection was split equally and processed accordingly. Our purification protocol captured almost 30 × more vector genomes per cm^2^, indicating equivalent titers may be gleaned from considerably less cell culture.

**Figure 4.**
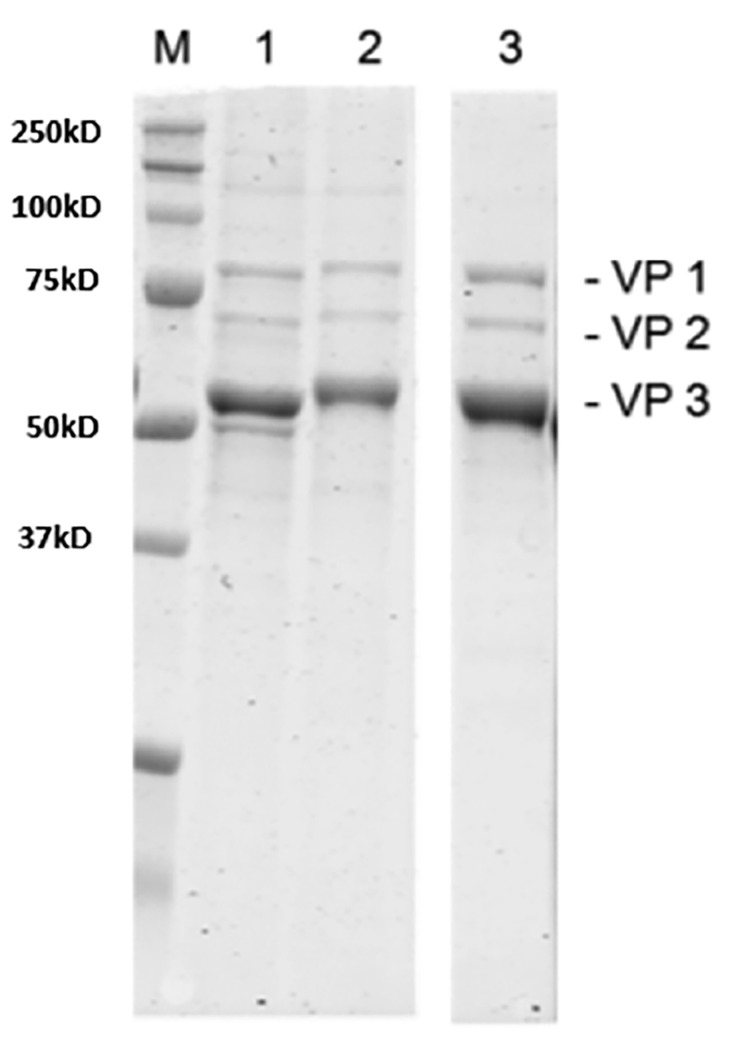
**SDS-PAGE electrophoresis of purified and suspended AAV2/6.** Lane: (M) Marker (Bio-Rad Precision Plus), (1) Viral pellet post 149,000 × g centrifugation suspended in 10ml buffer, (2) AAV2/6 viral suspension dialyzed overnight in suspension buffer to demonstrate further purification steps are not required and may result in titer loss, (3) heparin column purified AAV2/6 control, space indicates extraneous wells removed.

Aliquots at various steps of our protocol were collected to identify total protein loss and capture. Protein tracking experiments illustrate a step-wise progression to viral concentration and purity, and need not be performed for general quality control (QC) validation. To that end, we have instituted a three-tier QC process involving SDS-PAGE/Coomassie staining to assess capsid capture and purity, qRT-PCR to identify vector genome titer and a bioactivity transduction assay to ensure transgene expression. Because these assays are common and dependent on individual AAV applications, their protocols fall outside the scope of this article. Vector genome titers were not performed on protein tracking samples as the highly variable nature of media and buffers confounds qRT-PCR results. Figures two and three illustrate quantitated total protein tracking and visualization respectively. Taken together, these figures demonstrate most contaminating proteins are removed during the crude pellet and final centrifugation steps.

The final purity of the AAV suspensions is visualized in **Figure 4**. Gels were imaged using a Li-Cor Odyssey that provides Coomassie resolutions equivalent to silver staining. As observed, very little cellular proteins are carried over to the final suspension. Also, dialysis may be implemented if further purification is required. It should be noted that lane smearing and non-capsid bands are most likely partially denatured viral aggregates.

**Figure 5.**
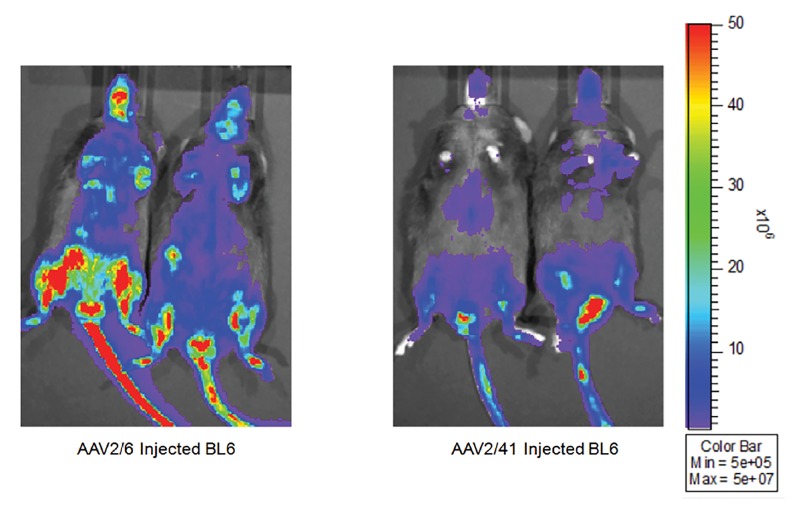
**Adult C57BL/6 mice imaged for luciferase subsequent to AAV2/6 & AAV2/41 systemic delivery.** Mice were tail vein injected with 2 × 10^12^ vg and imaged eight weeks after viral delivery. Our PEG/Spin protocol can successfully isolate functional naturally occurring and engineered AAV virions.

To confirm *in vivo* functionality, we delivered a CMV luciferase cassette to C57BL/6 mice at 2.0 × 10^12^ vg of AAV2/6 or AAV2/41 (**Fig. 5**). Virus was delivered systemically via tail vein. Both of these preparations successfully delivered the reporter cassette to murine tissues and demonstrated expression patterns consistent with published literature. Variable luciferase expression levels are attributed to different capsid infectivity efficiencies and are not reflective of our process.

In conclusion, this rapid PEG/Spin protocol for AAV purification produced significantly higher yields than traditional column chromatography. Both wild-type and bioengineered virions were able to be captured and functioned equivalent to column purified AAV. This protocol represents an inexpensive, efficient and scalable method for AAV purification in the laboratory setting that is independent of capsid serotype or moiety.

**Figure 6.**
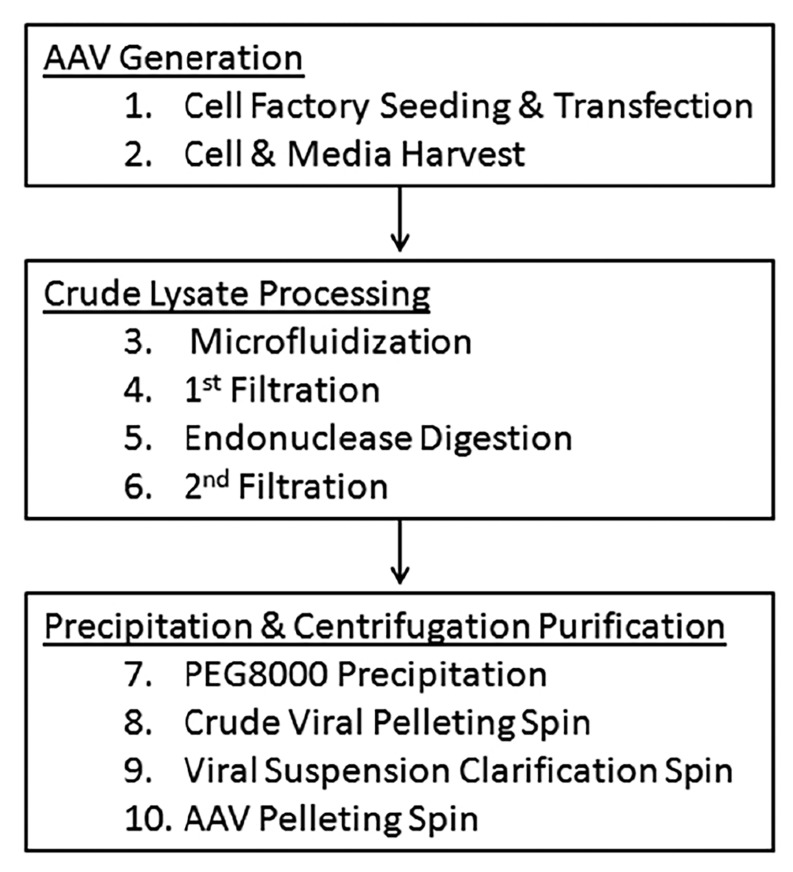
Work flow diagram outlining stepwise progression through our protocol.

## TROUBLESHOOTING

Possible problems and their troubleshooting solutions are listed in **Table 1**.

**Table 1.** Troubleshooting.

Step	Problem	Possible reason	Solution
1.8	Precipitate not visible	• pH of BBS solution is off • More NaPO4 required.	• Re-pH buffer • Spike mix with 1/100^th^ volume Hyclone 1x PBS
1.8 *(cont’d)*	Precipitate too large	• Contaminants in DNA • Incubation time too long • Mixing method not optimized.	• EtOH Clean-up/Re-purify DNA • Examine aliquots at closer time intervals • Use an alternative method for combining Transfection Mix A & B
4.9	Difficulty re-suspending, capsid complexing with nucleic acid	• High titer capsids aggregate with themselves and contaminants inhibiting accurate QC analysis and transduction. To assess complexing, purified samples may be analyzed on SDS-PAGE and agarose gels.	• Suspension of PEG/NaCl and final pellet must be carried out in high osmotic buffers to prevent aggregation.
